# COVID-19: Short-term forecast of ICU beds in times of crisis

**DOI:** 10.1371/journal.pone.0245272

**Published:** 2021-01-13

**Authors:** Marcel Goic, Mirko S. Bozanic-Leal, Magdalena Badal, Leonardo J. Basso

**Affiliations:** 1 Department of Industrial Engineering, University of Chile, Santiago, Chile; 2 Instituto de Sistemas Complejos de Ingeniería (ISCI), Santiago, Chile; 3 Department of Civil Engineering, University of Chile, Santiago, Chile; Fuzhou University, CHINA

## Abstract

By early May 2020, the number of new COVID-19 infections started to increase rapidly in Chile, threatening the ability of health services to accommodate all incoming cases. Suddenly, ICU capacity planning became a first-order concern, and the health authorities were in urgent need of tools to estimate the demand for urgent care associated with the pandemic. In this article, we describe the approach we followed to provide such demand forecasts, and we show how the use of analytics can provide relevant support for decision making, even with incomplete data and without enough time to fully explore the numerical properties of all available forecasting methods. The solution combines autoregressive, machine learning and epidemiological models to provide a short-term forecast of ICU utilization at the regional level. These forecasts were made publicly available and were actively used to support capacity planning. Our predictions achieved average forecasting errors of 4% and 9% for one- and two-week horizons, respectively, outperforming several other competing forecasting models.

## Introduction

The first cases of the COVID-19 pandemic were detected in Chile by early March 2020. A few days later, all schools were closed, and a few counties with relatively high numbers of cases were quarantined. By the end of April, the available data showed that the outbreak was kept relatively under control, with a few hundred confirmed new cases every day. However, by early May, the infection rate started to increase rapidly, threatening the ability of health services to accommodate all incoming COVID-19 cases. In the middle of May, the Chilean Society of Intensive Medicine (SOCHIMI) reported a worrisome occupation rate of ICU beds of more than 95% in the capital city of Santiago, where most of the cases were concentrated. Suddenly, ICU capacity planning became a first-order concern. On May 12th, the Instituto Sistemas Complejos de Ingeniería (ISCI), which was already working on analytics related to mobility, was urged to prepare short-term forecasts of ICU bed occupancy rates for those regions with the highest utilization rates. Within 24 hours, we submitted our first report. From then on, we prepared forecasts every two days for several weeks, and then we reduced the frequency and began reporting every four days. These reports were sent directly to the authorities –particularly those on the coronavirus response committee– and to SOCHIMI. Additionally, we published the reports on ISCI’s website (https://isci.cl/covid19/). The reports grew in complexity and regional coverage over time based on what decision makers deemed to be most pressing.

We developed a solution for generating predictions of the number of ICU beds that were going to be required by COVID-19 patients for every region in the country with a time horizon of 14 days ahead. Our methodology was based on an ensemble of a variety of forecasting models that capture different components of the evolution of the outbreak. The first model we built was a compartmental model that described patient flow as a stochastic progression through different clinical states. Here, we contemplated that new patients would require an ICU bed after a specific number of days with a given probability, and they would be discharged after a given number of days according to a certain distribution. Compartmental models have been some of the most popular approaches for characterizing the evolution of epidemics [1, 2], but they have limited flexibility to accommodate dynamic variations in the environment. In the context of COVID-19, the delay between the identification of a new case and the requirement of an ICU bed, the duration of mechanical ventilation, and the likelihood of requiring urgent care are some of the critical parameters that might change over time, and these are not properly captured by this kind of model. Therefore, we included several autoregressive and machine learning models that could better capture dynamic variations in the environment. Then, we combined the forecasts output by the different models using a trimmed mean ensemble. Our approach could generate accurate forecasts, achieving average prediction errors of 4% and 9% for one- and two-week horizons, respectively. These predictions were informative for supporting decision makers during the sanitary crisis, and our approach outperformed other competing ensembles of forecasting models.

In this article, we describe in detail the methodology we used to generate forecasts for this very urgent problem, showing how the use of analytics provided relevant support for decision making in critical times, even with incomplete data and without enough time to fully explore the numerical properties of all available forecasting methods. Using this methodology, we produced predictions with small forecast errors that not only were useful for supporting decision making in critical times but could also be informative with regard to resource planning for potential new outbreaks. Most importantly, our approach may be easily replicated in other countries facing acute capacity constraints with respect to ICU beds.

The rest of the article is structured as follows. In Section 2, we describe the context and the data we had available, and we provide some institutional background that imposed some constraints on the design of the forecasting methodology. In Section 3, we review the relevant forecasting literature, and then we describe the statistical models we used and how we combined them to produce our forecast. In Section 5, we discuss some adjustments we introduced to accommodate changing conditions in the spread of COVID-19 and present our forecasting results. Section 6 contains a nontechnical summary of the methodology, its results and its advantages over other approaches, and we discuss possible implementations in other parts of the world. Thus, Sections 2 through 5 are devoted to providing a comprehensive technical documentation of the underlying methods we used, while readers more interested in results and implementations may read Sections 2, 6 and 7.

The rest of the article is structured as follows. In the next section, we describe the context and the data we had available, and we provide some institutional background that imposed some constraints on the design of the forecasting methodology. In the following three sections, we present the technical elements of the forecast: (i) we review the relevant literature, (ii) we describe the statistical models we used and how we combined them to produce our forecasts, (iii) we discuss some adjustments we introduced to accommodate changing conditions in the spread of COVID-19 and (iv) we present our forecasting results. These sections are devoted to providing a comprehensive documentation of the underlying methods we used. Readers more interested in results and implementations can go directly to the nontechnical summary of the methodology and results, where we present a summary of the methodology, its results and its advantages over other approaches and discuss possible implementations in other regions. We conclude with a discussion on the implications of our findings and avenues for future research.

## The urgent problem of forecasting ICU beds

**Fig 1:**
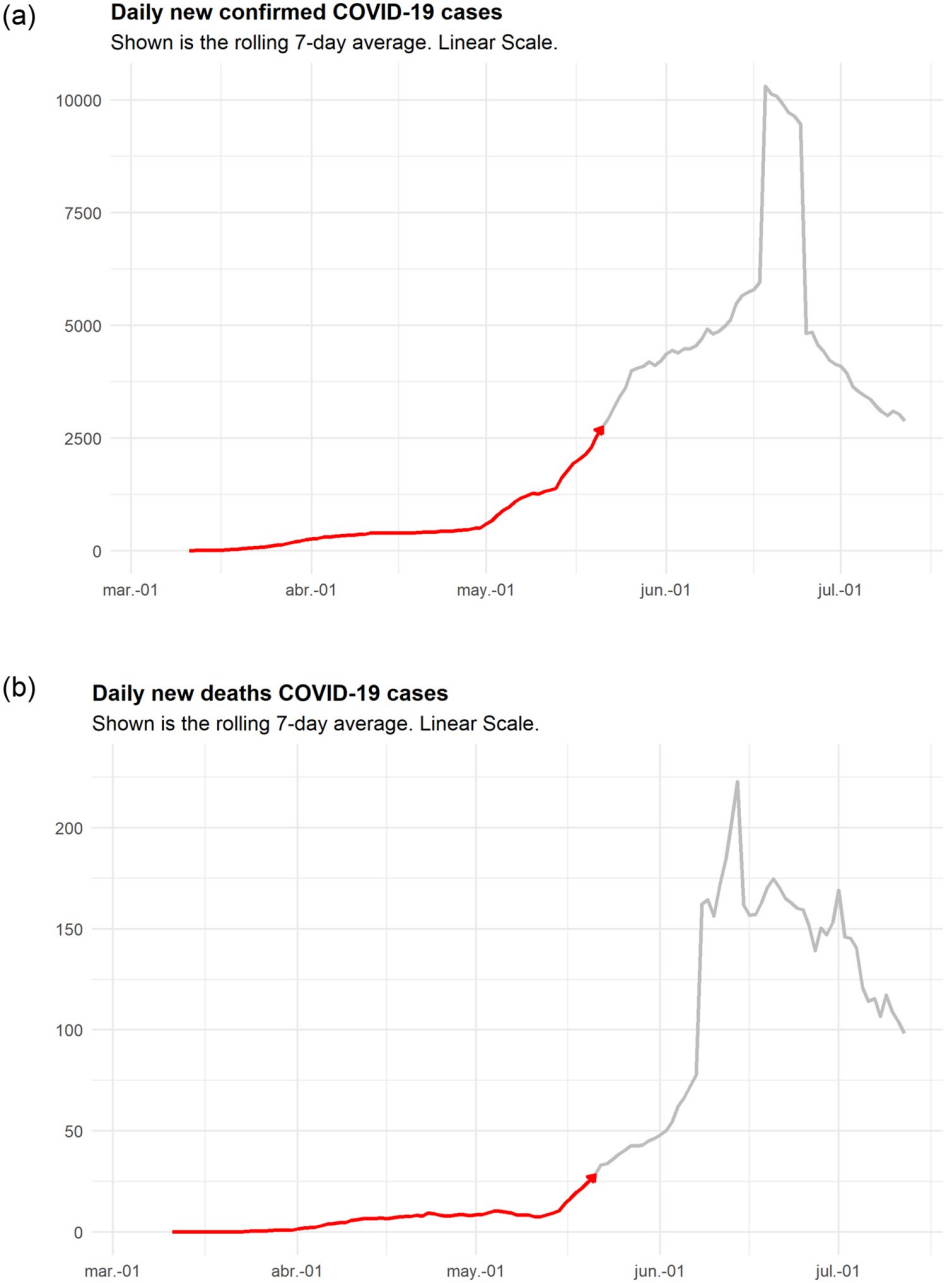
Evolution of new confirmed cases and new deaths from COVID-19 at the national level. The large peak of new cases registered in late June corresponds to a change in the governmental procedures to count cases, and this added a large number of cases that were not previously considered.

The first COVID-19 cases were detected in Chile by early March 2020, and for the first two months, the number of new infections was relatively under control, with a few hundred confirmed new cases every day. However, by early May, the number of new COVID-19 cases increased rapidly, creating numerous and complex challenges for the country. A graphical illustration of the evolution of the pandemic in Chile is displayed in Fig 1. In the left panel, we display the series of newly confirmed cases, and in the right panel, we display the series of new deaths. In both cases, we highlight the state of the series at the time we started producing the forecasts, which was a few days after the country entered a severe exponential growth phase. By looking at the international experience and learning from the problems faced by other countries that were affected earlier by the pandemic, it was clear that the management of hospital capacity was going to be a critical decision [3]. Furthermore, upon following the exponential trend of new cases, it was concluded that there was a serious concern that the capacity of ICU beds could be dramatically surpassed, leading to greatly increased mortality rates.

To increase ICU capacity, hospital management can follow a number of complementary strategies with different levels of complexity. A simple mechanism to increase hospital capacity is through the release of medical resources by rescheduling nonurgent procedures. Other strategies require more time for implementation. For example, pediatric rooms could be converted to receive adult patients, or anesthetic machines could be adapted to provide mechanical ventilation. As most of these mechanisms could be implemented within a time span of a few days, we decided to provide forecasts for a 14-day horizon. Despite generating forecasts of ICU utilization for each of those fourteen days, in the reports, we highlighted the number of beds that would be required in exactly one and two weeks.

Chile is administratively divided into sixteen regions, and in terms of geographical aggregation, forecasts were produced at the regional level. The country’s population is very unevenly distributed, and the Metropolitan Region, which includes the capital city of Santiago, contains near half of the national population. Despite this heterogeneous population distribution, our decision to produce regional demand forecasts is justified for two reasons. First, consistent with the administrative division, budgets are executed at the regional level. Second, from an operational perspective, if needed, patients can be transported from one hospital to another within the region, and therefore, the capacity at the regional level provides the most useful aggregation for decision making.

To estimate the models, we used data that were publicly available. Given the crucial importance of the consequences of the pandemic for the whole nation, the Ministry of Health provided frequent epidemiological reports starting on the day of the first infection. Later, the Ministry of Science consolidated all available information and created a public repository with an extensive list of statistics reported in a time series format (http://www.minciencia.gob.cl/covid19). The data series were reported at either the national, regional or county level, with only a few statistics available at a more disaggregated level. Throughout the whole process, we tried to include different covariates in the model, but the results we generated are based on the list described in Table 1. The main series we studied was the Regional Number of COVID-19 Patients in the ICU. The other series were used as additional explanatory variables.

**Table 1:** Publicly available data used in the forecasts.

	Aggregation		
	Since	Geographical	Time
Number of PCR tests	2020-04-09	Regional	Daily
Number of COVID-19 patients in the ICU	2020-04-01	Regional	Daily
Number of COVID-19 patients in the ICU by age group	2020-04-01	National	Daily
Number of new symptomatic cases	2020-03-03	Regional	Daily

At the beginning of our study, the repository had information on the total daily number of new infections by region, but a few weeks later, the repository started reporting the number of new cases while distinguishing between symptomatic and asymptomatic cases. As the latter did not require ICU beds, from then on, we decided to only consider the series of symptomatic cases.

With these data in hand, we embarked on the challenging task of producing demand forecasts for ICU beds. Certainly, accurate predictions could assist decision makers in effectively preparing for the large number of expected hospitalizations. However, the exponential nature of the infections generated large variations in the expected numbers of patients in different scenarios. As we were urged to do, our goal was to create robust predictions and deliver them to health officials, with the aim of supporting them with information that could help them understand the rate at which they should be increasing ICU capacity.

## Literature review

Our work is related to two streams of research. First, our research is related to the use of analytics in health care and, in particular, to the use of forecasting methods for planning healthcare capacity. Second, our research is related to the use of pooled forecasting and the combination of multiple methods to generate robust predictions. Next, we discuss both streams of literature with a special focus on other recent works in the context of COVID-19.

Analytics have been shown to be relevant for supporting decisions in different components of healthcare systems [4, 5]. In recent years, we have seen an explosive growth of analytics applications in diverse facets of health care, including medical diagnosis, human resources, supply chain management, and the design of health care insurance (to name a few) [6, 7]. Although the use of mathematical modeling in this area has brought a number of challenges, there are ample opportunities to generate essential and timely knowledge to support decision-making [8]. In the context of the control of infectious diseases, the combination of big data and tractable analytical techniques has provided new tools to fight against pandemics [9]. The global impact of COVID-19 has motivated numerous modeling efforts to provide guidelines for the control and management of the outbreaks. Certainly, there is a close relationship between the spread of the infection and the demand for medical resources. Therefore, mathematical models that describe the evolution of the pandemic can provide a first-order approximation of the demand for ICU beds. For this reason, we were especially concerned about the modeling effort needed to forecast the spread of the outbreak for the purpose of estimating the requirements of hospital resources. For example, [1, 2] used different nonpharmaceutical intervention scenarios in the UK and Italy, respectively. Similarly, [10, 11] evaluated the impact of mobility and traveling on the spread of the virus, while [12] assessed the effect of age structures on fatality rates.

Similar to our study, other works have proposed different models to forecast the number of infections. For instance [13], used logistic growth models and a sub-epidemic wave model, and [14] used autoencoders to provide short-term forecasts for the total cumulative and newly confirmed cases in several provinces of China. [15] used an exponential growth model that included recovery and fatality rates to analyze the evolution of the total number of cases in the US, Slovenia, Iran and Germany. Finally [16], introduced a state-space hierarchical model to generate short-term daily forecasts considering the relations between the series of different countries. These investigations have advanced our understanding of how the pandemic spreads, but they are silent about the use of hospital resources.

The use of forecasting methods to aid hospital resource planning has been an active area of research. In this regard, time-series analysis has been one of the most widely used approaches for generating short-term demand forecasts because it provides a comprehensive treatment for seasonality and serial correlations. For example [17], assessed the prediction accuracy of short-term emergency bed occupancy for different time-series methods and historical average models [18]. Analyzed different time-series methods to forecast emergency bed occupancy and showed that they can provide meaningful information up to one week ahead.

To predict medical requirements with a longer time horizon [19], proposed a seasonal ARIMA to characterize the volatility of a series. They found that the model produces good forecasts most of the time, but it breaks down during a crisis. On a different line of research [20], employed individual patient-level data in a computationally intensive model to forecast the demand for beds at different units in a hospital.

Since the start of the COVID-19 pandemic, there have been several attempts to estimate the demand for hospital resources. However, as most of this work is devoted to describing the aggregated evolution of such requirements, the results are useful for anticipating global policy making but not for supporting tactical decisions. For example [21], used simple Gaussian curve fitting to predict the number of ICU beds and mechanical ventilators used at the peak of the outbreak and the cumulative use of bed days. Highly complex epidemiological models have also been used extensively in this context. Cancino and Rainisch [22, 23] used compartment models with age structures to simulate the spread of the virus and evaluate the impact of mitigation strate-gies on the healthcare demand at the peak of the epidemic.

Similar to these investigations, for our predictions, we developed a compartmental model, but we tailored it to the prediction of the demand for ICU beds in the short term. To do so, we limited our attention to the progression of patients after they had been diagnosed, and we considered a parametric distribution for patients requiring an ICU bed. Here, we incorporated clinical parameters that describe the clinical evolution of patients, and we derived detailed predictions for critical medical resources. An important drawback of compartment models is that they have limited ability to accommodate dynamic changes in key parameters; therefore, their predictions may fail to capture important variations in a given process, such as congestion or delays in testing. To overcome this limitation, we relied on ensemble forecasting models, where we combined compartment model predictions with those derived from autoregressive and machine learning models; these can effectively capture dynamic variations in the environment.

To integrate different forecasting models, we used an ensemble approach. Previous works offered strong evidence supporting the idea that combining forecasts can improve the accuracy of the output predictions [24, 25]. The literature on pooled forecasting indicates that the simple average of predictions performs well, but in our case, we used a special form of the trimmed mean to accommodate some specific features of our problem [26].

Pooled forecasting has been applied in many diverse domains [27], but its applications in the context of the pandemic are scarce.. [28]. Combined a logistic growth model with machine learning predictions to estimate the epidemic curve and predict the overall trends of the epidemic [29]. Applied a wide variety of forecasting models, including autoregressive models, random forests, ridge regression and support vector regression, to provide very short-term forecasts of the cumulative number of confirmed cases in Brazil, and they compared the performances of individual models against an ensemble prediction. Similarly [30], used an optimization-based ensemble to find the best combination over a family of machine learning predictions and applied this methodology to predict the cumulative number of hospitalized patients in Andalusia. Following a different approach [31], used neural networks to extract features from time-series data and then used those features to feed standard compartment models for the purpose of describing the aggregated spread of the pandemic.

There are two main elements that differentiate our research from other works using multiple models. First, our method combines different predictions to produce robust estimations of the required number of ICU beds. These models can capture different components of the spread of COVID-19. For example, we considered machine learning models that can provide a great deal of flexibility to accommodate short-term variations in the environment, but we also included compartment models that provide a more detailed description of the clinical components of the disease. The second distinctive feature is that our approach is specifically devoted to supporting ICU capacity decisions; therefore, we tailored our predictions to estimate the number of beds that would be required at each point in time rather than only aggregated metrics, such as the number of beds at the peak or the cumulative number of beds that would be required over the whole duration of the pandemic.

## Models

The most widely used approach for describing the evolution of infectious diseases is compartmental models, where the population dynamically evolves through different stages [2, 32, 33]. In one of the simplest versions, healthy (and susceptible) people become infected when they are in contact with an infectious individual, after which they eventually recover or die. These models have been extended to include clinical stages, providing a first approximation for the use of hospital resources [34, 35]. In addition, as compartmental models directly describe the dynamics of the disease, they can be suitable for guiding the evaluation of mitigation policies [1]. Nonetheless, these models require precise estimations of the relevant epidemiological and clinical parameters, which have been proven to be difficult to estimate in practice [36, 37]. Furthermore, for the specific problem of forecasting the COVID-19-related demand for ICU beds, we had good reasons to believe that several of the key parameters could change rapidly over time, generating biased predictions. We identified at least three reasons why epidemiological parameters could be nonstationary:

1. The proportion of symptomatic patients requiring mechanical ventilation can change over time, and similarly, clinical criteria for releasing patients from the ICU can be adjusted dynamically depending on the actual usage of the existing capacity. This is not only because hospitals can relax nominal criteria but also because SARS-CoV-2 is a new virus that involves continuous learning by medical teams. For instance, the head of the Chilean Society of Intensive Medicine stated that “Patients initially stay in the ICU between 10 and 11 days, and now they are staying between 14 and 16 days. This is because, with everything we learned, we intubated less and selected more serious patients“(https://bit.ly/2BTAuJK).
2. Despite governmental efforts to provide timely access to relevant information, a large portion of the system for generating the data was under constant stress, and therefore, the information that we had available for any single day could be lagged. Among other issues, the results of lab tests exhibited important delays worldwide (https://www.usatoday.com/story/news/health/2020/07/11/COVID-19-test-results-delayed-labs-struggle-cases-surge/5406936002/); hence, the number of new cases might be more or less informative depending on the congestion of the laboratories.
3. The data were not always available at the patient level, and there are important factors that were never observed. For example, every day, the government reported the number of new cases and the current occupation of ICU beds per region, but there was no information about how many patients entered or exited or on the lengths of the stays of patients in intensive care. Likewise, when the capacity was lacking in some regions, the government had the ability to move patients between regions, and this was not systematically reported.

To overcome the limitations of compartmental models and properly capture short-term dynamics, we combined these models with other time-series models that could be better suited to capture those dynamics. From a theoretical point of view, the use of combinations of forecasts is justified because they can lead to smaller forecasting errors and can even reduce the biases of individual forecasts [38–40]. Beyond theory, combined forecasts have been shown to lead to improved performances in a wide range of applications [25, 27, 41].

Previous studies have offered several reasons to justify the empirical success of combining forecasts; these include model misspecification, changes in the underlying parameters and the heterogeneous use of different information sets [42]. As we have explained, several of these reasons were present in our setting, and consequently, our general approach was based on an ensemble of different forecasting models. Next, we briefly discuss the individual models we included in the ensemble. To organize the discussion, we group the list of models into three categories: autoregressive models, artificial neural networks and compartment models.

### Autoregressive models

#### ARIMAX

We start with a classic autoregressive integrated moving average (ARIMA) approach [43]. In this model, the values of the time series on day *t* (*y*_*t*_) depends on their lagged values and its lagged errors, and the series are further differentiated to estimate stationary processes. The ARIMAX variant is the result of considering an additional set of exogenous explanatory variables *x*_*t*_. In the vector *x*_*t*_, we considered the whole series of new cases and the positivity rate. By introducing the backward shift operator *B*, the model can be expressed in a compact form as:

$$\begin{array}{cc} \phi_{p}\left(B\right){\left(1-B\right)}^{d}y_{t} & =\beta^{\prime }x_{t}+\theta_{0}+\theta_{q}\left(B\right)\varepsilon_{t} \end{array}$$ (1)

The model depends on the relative weight of its own values (*ϕ*), the weights of the errors (*θ*), the weights of the exogenous variables (*β*), and the constant term (*θ*_0_). The model also depends on the number of lags (*p*, *q*) and the number of difference operations (*d*). To determine the value of (*p*, *d*, *q*), we used stepwise selection based on the AIC [44].

In our analysis, we considered ARIMA and its ARIMAX variants, but the forecasts of both were fairly similar; therefore, to create our ensemble forecast, we only considered one of the two. For ARIMAX, we included the number of new symptomatic infections in previous days as one of the key explanatory variables. For more flexible models, we considered the whole sequence of new symptomatic infections; in this case, we only included a few values in the [6–12] range that were shown to provide more stable estimates than those obtained by feeding the model with the complete series.

#### TBATS

We then looked at a trigonometric seasonality, Box-Cox transformation, ARMA errors, and trend seasonal components (TBATS) model. This model uses a combination of exponential smoothing and Box-Cox transformations to automatically accommodate multiple seasonal components. Each of these seasonalities is modeled by a trigonometric representation based on a Fourier series. Although this model considers a series of nested equations to represent a detailed decomposition of the series, using the backward shift operator, the model can also be expressed in a reduced form as:

$$\begin{array}{cc} \phi_{p}\left(B\right)\eta \left(B\right)y_{t}^{\left(\omega \right)} & =\theta_{q}\left(B\right)\delta \left(B\right)\varepsilon_{t} \end{array}$$ (2)

Here, $y_{t}^{\left(\omega \right)}$ is the Box-Cox transformation of the series. The operators *η*(*B*) and *δ*(*B*) are reduced-form expressions summarizing local levels, short- and long-term trends and the sequence of seasonality components. For a description of the extensive form of this model, see [45].

One of the advantages of this model is that it provides a great deal of flexibility to automatically accommodate a large number of seasonal and trend components. However, unlike the previously discussed ARIMAX model, TBATS does not include exogenous variables and hence has limited ability to anticipate how variations in infections can be translated into different requirements of ICU beds.

### Time-delay artificial neural networks

In our approach, we included several neural network models. To accommodate the time series structure, we used a special class called time-delay neural networks (TDNNs). In this class, the inputs to any node can include outputs of earlier nodes not only during the current time step but also from previous time steps [46].

As is common in neural network learning, we trained the model structure by adjusting its parameters to minimize the induced error using a generalized feed-forward network. Thus, without loss of generality, the predictions are given by:

$$\begin{array}{c} y_{t}=g(\sum_{j=0}^{q}v_{j}f(\sum_{i=0}^{p}w_{ij}\phi_{i}\left(y_{t-i},x_{t-i}\right))) \end{array}$$ (3)

In this expression, *f* is the activation function for the hidden layers, and *g* is a nonlinear transformation in the output layer. Additionally, the *ϕ*_*i*_(*x*) are the basis functions, and (*v*, *w*) is the list of weights that are calibrated during the training process. Neural networks have been shown to exhibit superior forecasting power to other methods in different settings [47]. The TDNN models we present next vary in their structures (numbers of input nodes *p* and hidden nodes *q*), as well as in the pruning criteria used to reduce the dimensionality of the network and avoid overfitting.

#### MLPR

A perceptron is a classifier that maps a vector of inputs to a single binary value through a threshold activation function. A multilayer perceptron is a network of individual classifiers that enables learning about complex processes, and it is one of the most commonly used perceptron-based learning algorithms [48]. The flexibility of an MLPR algorithm allows for the inclusion of an arbitrary set of input variables, such as the lagged values of the series and other explanatory factors. In our implementation of MLPR, we followed the general expression of (3) with logistic activation functions and four hidden layers. We tried different numbers of nodes per layer but ended up using a {5:10:10:5} architecture, which performed well.

#### ELM

An extreme learning machine is a special feed-forward neural network that only uses a single hidden layer. In this layer, nodes are randomly chosen, and the weights of the outputs are analytically determined [49]. Unlike those of other back-propagation learning algorithms, the parameters of the hidden layer of an ELM do not need to be tuned. In fact, an ELM aims to not only minimize the training error but also to reduce the norm of the output weights. Thus, ELM models tend to achieve good generalization performances with much faster training processes than those of other artificial networks [50].

We implemented an ELM following the general TDNN expression in (3). The norm of the output weights was controlled by the LASSO, where the norm of the weights was imposed to be smaller than a given threshold. We decided to use 11 nodes in the single hidden layer because that resulted in good performance and small prediction errors.

#### GMDH

A “group method of data handling” approach involves the successive selection of models based on external prediction criteria. Starting with a simple set of models, the method constructs new generations of increasingly complex models and combines them to maximize the forecasting performance [51]. In our case, we organized the sequence of models in a neural network, where each layer corresponds to a new generation of models. Following other GMDH applications in the literature, we considered polynomial models as follows [52]:

$$\begin{array}{c} y_{t}=a+\sum_{i=1}^{m}b_{i}y_{t-i}+\sum_{i=1}^{m}\sum_{j=1}^{m}c_{ij}y_{t-i}y_{t-j} \end{array}$$ (4)

The GMDH method allows for the inclusion of an arbitrary set of covariates in the polynomial, but in the context of time series, we only considered the lagged values of the series. Our motivation to include this model in the pool of forecasts was that it was conceived to learn complex relationships when lacking detailed knowledge about the fundamentals of the given process. In our case, we had epidemiological theory characterizing the evolution of the pandemic, but the observed data were mediated by a number of unobservable processes that might require additional layers of complexity. Another strength of GMDH is that recent computational implementations of the algorithm include automatic normalizations of the variables [53].

### ICD compartment model

The goal of our compartment model is to predict the future utilization of ICU beds by critically ill patients due to cases of COVID-19. Thus, our model aims to replicate the behavior of the ICU process, balancing inbound and outbound flows of patients in different stages of the process. Our model considers three compartments through which the patients evolve. For each of them, we tracked the number of patients in each stage as follows:

- I: The number of *infectious* individuals who show symptoms of COVID-19.
- C: The number of *critically ill* people who need an ICU bed.
- D: The number of individuals who are *discharged* from the ICU.

**Fig 2:**
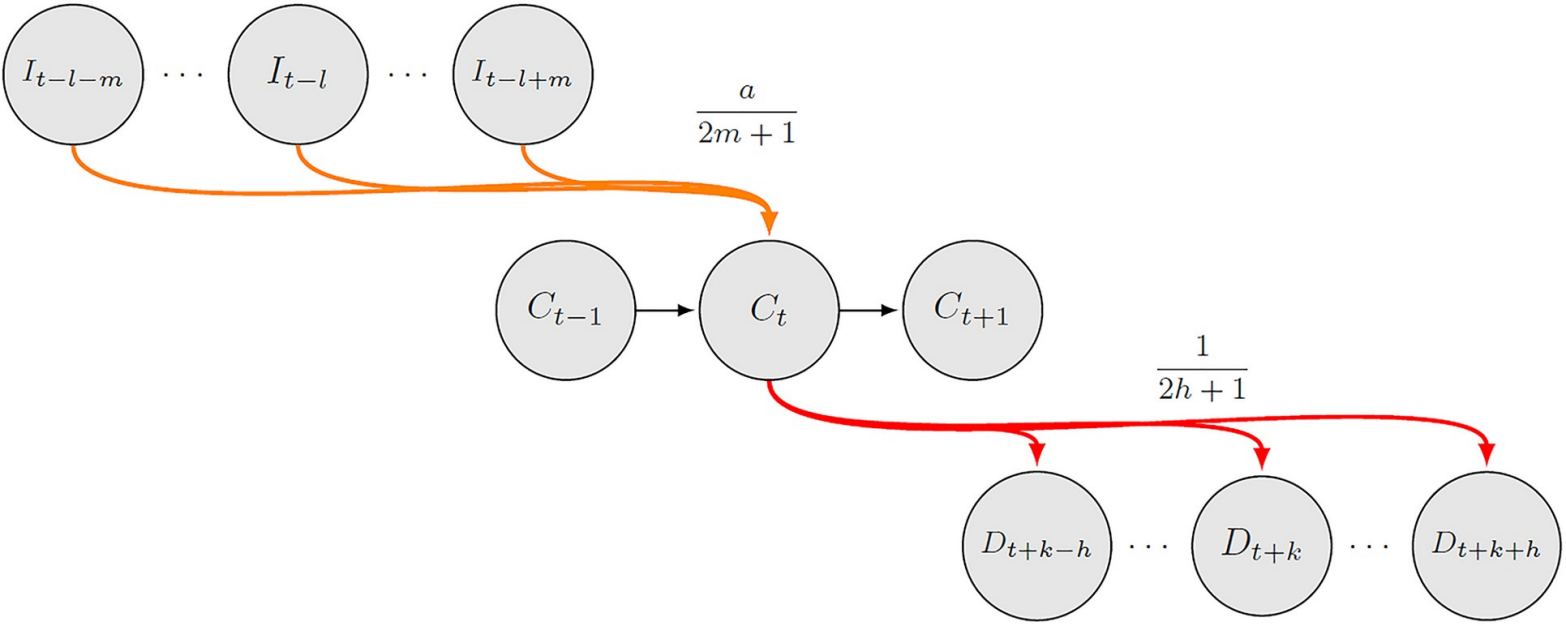
Compartment model diagram.

The number of infected, critically hospitalized and discharged patients fluctuated over time; therefore, we made the state variables dependent on time. Thus, the variables *I*_*t*_, *C*_*t*_ and *D*_*t*_ represent the number of new symptomatic cases, the number of critical patients and the number of discharged cases on day *t*, respectively. We describe the transitions between states using a probabilistic approach. These probability distributions consider not only how likely it is that a given patient evolves to another state but also the expected duration of that transition, as illustrated in Fig 2.

Fig 2 illustrates that the number of ICU beds that will be used on day *t* depends on the number of beds used the previous day, the number of new cases that require critical care and the number of patients that will be discharged. Here, a fraction *a* of the symptomatic cases require an ICU bed, but they only demand beds *l* days after they are diagnosed. Acknowledging that there are variations in the delays since the diagnoses, we considered that patients requiring a new ICU bed on day *t* could have been diagnosed between *l* − *m* and *l* + *m* days before that day. While our model allows for any arbitrary distribution to characterize the requirements of beds over time, in this work, we only considered uniform distributions; therefore, a fraction $\frac{a}{2m+1}$ of new symptomatic cases detected on *t* − *l* will require an ICU bed on *t*. The logic for discharging patients is similar, but we know that sooner or later, all patients will be discharged; therefore, all uncertainty is associated with the duration of bed usage. We assumed that on average, patients are discharged after *k* days, but as before, we allowed for dispersion and let patients complete their clinical cycle in the [*k* − *h*, *k* + *h*] range. If the lengths of stay were uniform, the fraction of patients entering the ICU on day *t* who were discharged within time *t* + *k* was $\frac{1}{2h+1}$. In the empirical application, we used a bimodal distribution with a fraction *d* of moderately severe cases staying in the ICU between *k*_1_ − *h*_1_ and *k*_1_ + *h*_1_ days and more severe cases staying between *k*_2_ − *h*_2_ and *k*_2_ + *h*_2_ days. Formally speaking, the equations describing the evolution of patients over time are given by:

$$\begin{array}{c} C_{t}=C_{t-1}-D_{t}+\frac{a}{2m+1}\sum_{i=t-l-m}^{t-l+m}I_{i} \end{array}$$ (5)

$$\begin{array}{c} D_{t}=\frac{a}{2m+1}\cdot (\frac{d}{2h_{1}+1}\sum_{j=\left(t-l-m\right)-k_{1}-h_{1}}^{\left(t-l+m\right)-k_{1}+h_{1}}I_{j}+\frac{\left(1-d\right)}{2h_{2}+1}\sum_{j=\left(t-l-m\right)-k_{2}-h_{2}}^{\left(t-l+m\right)-k_{2}+h_{2}}I_{j}) \end{array}$$ (6)

In these equations, the series of ICU utilization and the number of new symptomatic cases are the data, while (*a*, *d*, *l*, *m*, *k*_1_, *h*_1_, *k*_2_, *andh*_2_) are parameters to be estimated. These parameters are disease-specific, and we could retrieve their values from the medical literature on SARS-CoV-2. For example, the mean duration of symptoms before hospital admission was reported to be 10±2 days [54, 55]. Similarly, medical reports have indicated that the length of stay depends on how severe the disease manifests in the patient, and it is estimated to be 21±7 for those who show extremely severe symptoms and 14±3 for those who do not [55]. In our model, we used these clinical estimates as references, but we conducted an exhaustive search over a grid centered around those values to choose the parameters that minimized the forecasting error for a holdout sample.

### Ensemble

An extensive body of literature has shown that combining forecasts can improve prediction accuracy and that a simple average often performs better than highly complex combination schemes [56]. However, as the mean can be sensitive to extreme values, recent studies have suggested that deleting the most extreme predictions might further improve pooled forecasting. For example, the median forecast might be less susceptible to being affected by outliers than the mean forecast [24].

In our application, we used a trimmed mean approach [26], where we used the simple forecast mean after discarding the two most extreme predictions. We introduced two variations to this procedure to accommodate our forecasting needs. First, as the predictions of ARIMA and ARIMAX were highly correlated, in the pool of selected forecasts, we only considered at most one of them. Second, as the medical personnel in charge of facilitating new ICU beds had a highly intuitive interpretation of the ICD model, we always included it in the pool. To rank the forecasts, we considered the prediction of ICU beds over a two-week horizon. Thus, if $y_{t}^{\left(k\right)}$ was the *k*-th order statistic for the series, then our forecast was given by the following trimmed mean:

$$\begin{array}{cc} y_{t}^{trim} & =\frac{1}{n-2}\{y_{t}^{ICD}+\sum_{k=2}^{n-1}y_{t}^{\left(k\right)}\} \end{array}$$ (7)

Thus, our forecast was composed of an average of four models (including ICD). Considering that these predictions directly inform health officials about critical decisions, we visually inspected all forecasts before producing the final reports. In these inspections, in very exceptional cases, when more than one forecast dramatically deviated from the mean, we overruled our trimmed criteria and included both ARIMA and ARIMAX in the forecasting pool.

### Implementation

When inspecting the series, we found no evidence of seasonality for any variable; therefore, all models were estimated using no seasonal components. To determine the number of observations to use in every forecast, we considered information starting from April 1st, when the accumulated number of symptomatic patients reached three thousand cases. Later, when more data were accumulated, we only considered the previous sixty days of data to estimate the models.

All models were estimated using daily data. During the pandemic, the Ministry of Health provided an updated report on the evolution of the most critical variables, such as the number of new infections, the positivity rate and the number of fatal cases. All this information is uploaded to the public repository of the Ministry of Sciences and Knowledge, from which we downloaded the information automatically. The data presented very few missing values, and to address them, we used a Kalman smoothing approach [57]. The parameters were independently calibrated for each model.

To determine the optimal values of (*p*, *d*, *andq*) for the ARIMA and ARIMAX models, we proceeded iteratively. If the value *d* was known, the model selected the orders of *p* and *q* via the AIC. For nonseasonal data, *d* was selected based on the successive KPSS unit-root test [58], which stops when finding a nonsignificant result [44]. In the case of TBATS, the general model considered several components; therefore, several variations were estimated (e.g., with and without trends, with and without Box-Cox transformations), and the final model was also chosen using the AIC [45].

Models based on artificial neural networks can be estimated using standard back-propagation learning algorithms. However, given the time-series structure, the estimation process benefits from using automatic feature selection [59]. For the case of GMDH, the weights of the polynomial were calibrated using a regularized least squares estimation method (RLSE), thereby reducing the potential problems of multicollinearity [53].

In terms of computational tools, data aggregation and preprocessing were conducted using R libraries. To normalize the data, we used Z-scores for the neural network models and Box-Cox transformations for the artificial neural networks and autoregressive models. When available, we use predefined libraries with forecasting methods. A table with the specific functions and parameters we used to implement each forecast is available in the S1 Table. For the compartment model and the ensemble, we coded our own routines to accommodate the specific requirements of the problem.

## Timeline of events and methodological adjustments

In the previous section, we described the general methodology we employed to produce daily forecasts for ICU beds. However, a key premise of this work is that the situation required urgent predictions. Moreover, the general environment was constantly changing, and therefore, we had to continuously update our methodology to accommodate the evolution of the pandemic and the information needs of health officials. The following is the list of the most relevant events that required adjustments to the methodology.

- We generated our first solution only a few hours after the government realized that ICU planning was going to be a key element in mitigating the consequences of the pandemic. These early solutions only considered reduced-form models with no epidemiological considerations. However, we quickly realized that we needed to complement these models with others that could capture the medical structure of the problem. This is because a large fraction of the decision makers who were actively reading our reports were healthcare professionals who needed a medical narrative to explain the variations in the demand for ICU beds. This narrative was only provided by a compartment model, and therefore, in all public reports we generated, we always included those models. We also tried using a linear regression model that could provide intuitive results; however, we found that for our particular case, the linear regression model had low predictive power.
- During the first two weeks, we used the series of newly confirmed cases regardless of whether the patients exhibited symptoms since that was the only information readily available. For the prediction of ICU beds, only patients with symptoms have a positive probability of requiring intensive care; therefore, the number of cases with symptoms should provide the most direct signal of the requirement for ICU beds. When the series of new cases was systematically reported depending on the existence of symptoms, we started to use symptomatic cases only.
- The first two reports we generated only considered the Metropolitan Region because it contained the largest number of cases by far; consequently, it was the most urgent concern for local authorities. After a week, we added reports for three other regions (Tarapacá, Antofagasta and Valparaiso) that also showed an alarming rise in new cases. At this point, our model was completely automated to generate predictions for all regions in the country, but we only progressively added more regions as they became more worrisome. By early July, we started reporting forecasts for all sixteen regions of the country.
- The GMDH model was not considered in the original list of models and was only introduced on June 11th. Since then, this model was been considered in the ensemble.
- In early July, we identified that most models were starting to show that the rate at which additional ICU beds were going to be needed for the Metropolitan Region was somewhat slowing down. However, the ICD compartment model did not show any sign of saturation. After interviewing medical personnel, we realized that some patients were starting to be mechanically ventilated in emergency rooms (ERs), and so they were not counted in the nominal series of ICU utilization. Thus, in terms of capacity planning, we were required to report how many beds should be made available to cover both new cases and ventilated cases in emergency rooms. Therefore, we complemented the series of ICU beds with the number of patients ventilated in ERs. Notice however, that the number of patients ventilated in ERs decreased to almost zero by late July, and therefore, we did not report them in the final two reports.
- As laboratories reached their testing capacities, the variation in the number of reported new cases increased significantly in mid-June. As a consequence, the forecasts were less stable. To overcome this problem, we preprocessed the series of new cases and used a five-day moving average instead of the raw series.

The results that we present in the next section are devoted to representing what we reported at each point in time, and they already include all methodological changes we introduced during the process.

## Results

Starting from May 16th, we generated standardized and frequent reports containing the two weeks ahead forecasts. The reports were made publicly available at https://isci.cl/covid19/ and were generated regularly every other day, except for the last two weeks of July, when the reports were generated only twice a week. The first reports only provided forecasts for the most critical regions, but we later provided reports for the whole country. In the analysis we present here, we only consider results since May 20th, when our routines were fully automatized to generate predictions for all regions.

**Fig 3:**
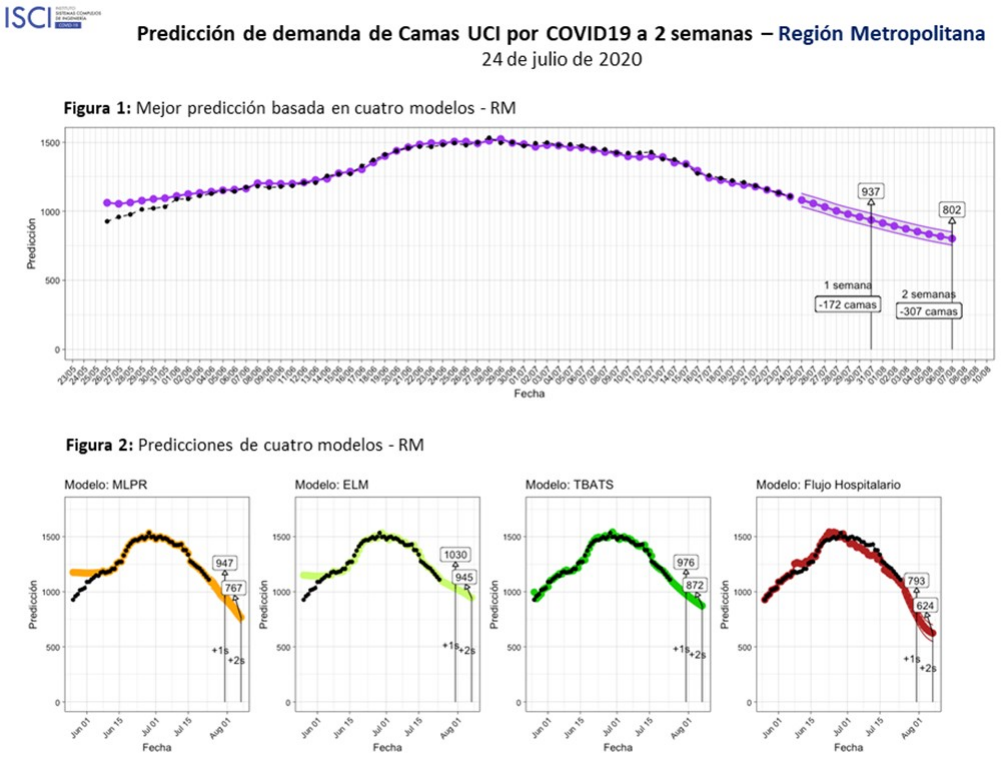
Government report: 24 July—Metropolitan Region.

The main body of each report consisted of a summary of the number of beds that were going to be required for each region for a time horizon of two weeks, followed by a graphical summary of the forecast. A very important requirement for these reports was that they had to be concise and easy to read. The crisis committee had a very short time to evaluate all the information, so our reports were tailored to consider this situation. In Fig 3, we display the predictions reported for the Metropolitan Region on July 24th. Graphical reports for two other regions and the national summary are available in the S1 Fig.

At the bottom of this figure, we show the forecasts provided for the models that survived the removal of the most extreme predictions, and then, in the upper part, we present the combined forecast. For all models, we presented both the predictions and the actual series of ICU occupancy. Furthermore, to facilitate the interpretation of the results, we highlighted the predicted numbers of beds that would be required in exactly seven and fourteen days. For the example presented in Fig 3, the reports indicated that the Metropolitan Region was going to require 937 beds within a week (172 beds less than occupation at that date) and 802 beds within two weeks (307 beds less than the occupancy on that date). For this particular example, the ensemble was produced with the MLPR, ELM, TBATS and ICD models, but those changed depending on the values of the forecasts. For a detailed count of the frequency with which each model was used in the ensemble, see the S3 Table.

For a systematic evaluation of our forecasts, we decompose the analysis into two parts. We first compare the performance of each model and the ensemble in terms of their forecasting errors, and then we discuss how our ad hoc trimmed algorithm fares against other pooling criteria.

### Model evaluation

**Fig 4:**
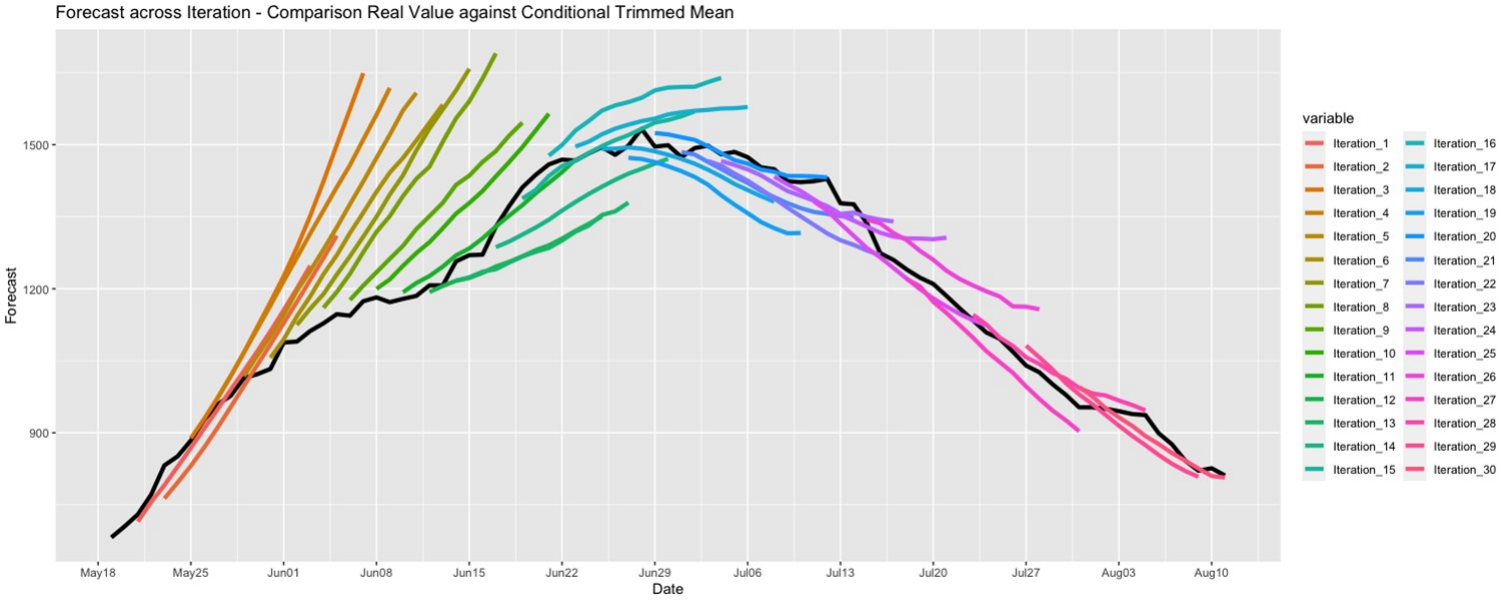
Forecasting iterations—Accuracy performance (ensemble).

From May 20th to July 28th, we produced 30 ICU utilization reports. In each report, we presented daily forecasts of the demand for ICU beds for the next two weeks in the regions considered in that instance. In every case, we generated predictions for different forecasting models, and we built our *best guess* through a conditional trimmed mean ensemble. A visual representation of all forecasts we reported for the Metropolitan Region is displayed in Fig 4. In this figure, we display the actual series of ICU occupancy with a black line, and each of the thirty fourteen-day ahead forecasts is presented with a different color. These results indicate that except for a few cases in early June, when we overestimated the demand for ICU beds, the predictions were, at least visually, quite accurate.

To summarize all these daily forecasts, we compute the mean absolute percentage error (MAPE) for each model, as displayed in Table 2. To simplify the exposition, we only report the performances of the models for the Metropolitan Region because this region required by far the most ICU beds in the country. For example, at the peak of the outbreak, the Metropolitan Region demanded more than 11 times more beds than the second-most congested region. An analogous table illustrating the errors for the Valparaiso Region (the second largest) is available in the S2 Table. Further metrics for other regions are available upon request.

**Table 2:** Historical MAPE per model—Metropolitan Region.

1st week								2nd week						
Date	Ensemble	ARIMAX	MLPR	ELM	TBATS	GMDH	ICD	Ensemble	ARIMAX	MLPR	ELM	TBATS	GMDH	ICD
2020-05-20	2.34	4.79	2.37	6.64	1.38		4.21	5.59	14.70	15.45	4.19	7.36		22.24
2020-05-22	5.15	4.68	7.21	8.21	7.63		6.86	6.95	4.00	3.03	9.91	3.30		37.49
2020-05-24	4.94	1.86	0.96	2.60	2.27		19.32	25.09	5.65	5.56	2.46	10.07		85.79
2020-05-26	8.64	4.42	3.33	4.68	4.29		21.55	25.57	14.51	12.13	16.99	14.21		57.76
2020-05-28	7.22	2.70	4.00	2.54	3.35		19.94	24.28	11.09	12.48	10.92	12.47		62.65
2020-05-30	7.00	2.94	2.91	2.24	2.54		21.41	22.99	12.75	6.50	10.59	10.38		62.11
2020-06-01	8.06	6.91	6.98	5.83	5.21		12.52	24.63	18.97	23.78	15.78	14.95		40.01
2020-06-03	9.74	3.80	7.74	3.74	3.33		23.81	23.62	11.02	14.57	9.50	8.06		59.39
2020-06-05	7.26	6.85	3.65	2.79	3.80		17.71	12.41	11.43	9.10	3.01	4.15		29.63
2020-06-07	6.31	4.40	9.38	3.19	2.83		14.80	7.64	4.75	19.77	2.56	3.11		27.66
2020-06-09	1.87	1.19	3.08	1.19	1.84		7.75	1.75	2.94	1.68	6.97	10.85		16.39
2020-06-11	3.70	2.88	6.45	4.58	5.31	1.53	2.46	10.23	5.75	16.44	12.22	14.99	5.41	7.96
2020-06-13	6.60	4.97	8.12	5.33	7.07	4.40	5.88	10.02	5.36	13.78	7.88	12.87	7.24	5.65
2020-06-16	6.31	1.39	7.93	5.69	6.29	7.42	6.36	4.27	7.78	8.26	3.65	5.24	5.36	2.37
2020-06-18	0.94	2.66	1.99	1.42	2.92	1.55	2.02	2.68	13.38	10.09	6.55	14.31	2.61	7.82
2020-06-20	4.32	6.09	7.37	3.44	6.60	2.56	4.36	8.03	18.85	14.82	10.91	20.00	8.65	2.66
2020-06-22	2.43	5.23	3.85	3.56	4.73	1.47	2.54	5.55	18.36	14.94	12.19	14.54	6.46	13.91
2020-06-24	0.90	1.64	7.37	5.38	1.24	3.13	6.58	3.35	7.20	20.61	17.28	4.10	12.59	35.56
2020-06-26	3.18	2.23	3.28	4.66	1.17	3.77	10.29	7.23	10.52	2.99	17.21	1.63	12.91	41.43
2020-06-28	1.25	8.03	3.12	9.17	6.23	7.87	12.23	0.66	20.76	11.03	23.01	15.49	19.75	44.11
2020-06-30	2.34	6.14	2.71	5.83	1.03	4.75	19.30	3.49	19.42	5.20	19.37	4.39	13.76	49.93
2020-07-02	3.04	5.71	1.85	7.26	1.08	4.77	21.82	4.94	19.64	14.47	23.15	5.77	14.10	57.32
2020-07-03	1.58	3.96	3.04	8.26	3.18	5.70	21.35	3.07	13.49	17.54	25.09	10.95	16.14	56.06
2020-07-07	2.00	1.03	6.85	6.30	1.67	5.81	22.81	5.01	9.92	27.33	27.88	11.99	20.55	50.17
2020-07-10	3.05	2.99	4.66	7.71	3.55	10.20	24.43	1.44	12.19	21.93	29.64	13.59	25.77	53.79
2020-07-14	4.00	6.69	9.56	12.81	7.02	16.80	19.80	8.16	14.84	23.18	33.07	16.38	33.26	54.20
2020-07-17	2.69	6.02	1.77	4.62	1.31	12.37	23.20	4.72	12.84	5.84	17.07	1.50	25.17	53.88
2020-07-21	1.37	6.91	2.04	9.47	4.88	14.59	14.72	2.67	13.29	5.99	18.57	7.53	32.82	38.47
2020-07-24	2.50	3.70	3.07	7.89	4.59	10.97	7.46	3.73	4.27	4.07	10.01	2.72	25.32	23.36
2020-07-28	2.59	1.60	3.39	4.51	2.11	9.64	4.56	1.26	2.74	6.74	10.34	2.76	29.24	3.47
min	0.90	1.03	0.96	1.19	1.03	1.47	2.02	0.66	2.74	1.68	2.46	1.50	2.61	2.37
max	9.74	8.03	9.56	12.81	7.63	16.80	24.43	25.57	20.76	27.33	33.07	20.00	33.26	85.79
mean	4.11	4.15	4.67	5.38	3.68	6.80	13.40	9.03	11.41	12.31	13.93	9.32	16.69	36.77
std	2.52	2.01	2.54	2.65	2.04	4.54	7.75	8.28	5.45	6.95	8.34	5.23	9.73	22.24

In Table 2, we further decompose the performance metrics into two blocks corresponding to the prediction vectors for 7 and 14 days ahead. It is expected that the predictions for the first week should have smaller errors than those of the second week. This is because more distant forecasts are associated with greater volatility and more degrees of freedom to drift away from the current value. The results from Table 2 confirm this idea and indicate that on average, our forecasts exhibited a 4.11% error rate in the first week and a 9.03% error rate in the second week. We believe these figures demonstrate that our forecasting approach provided good accuracy regarding capacity planning information in the context of the COVID-19 pandemic. Our view is confirmed by the assessment of the Minister of Science, who stated that our reports were “tremendously important to support decision making in difficult times“.

It is important to evaluate the performance of our forecasting approach in the context of a pandemic characterized by phases of exponential growth that can lead to large prediction errors. For example, consider the case of the U.K., where early epidemiological models initially projected approximately 500,000 deaths, a number that was updated to under 20,000 deaths just two weeks later [31]. For an additional discussion regarding the challenges in predicting the spread of COVID-19, see [60].

**Fig 5:**
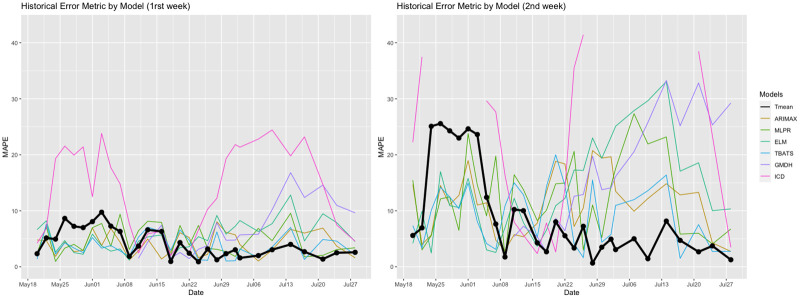
MAPEs across time for each iteration.

To further understand how individual models performed relative to the ensemble, we plot the series of MAPEs for all models in Fig 5. From this figure, we observe that the precision rates of the models are not uniform over time. With the exception of ICD, all models performed well early in the process and at the end, where the pandemic was either steadily on the rise or in decline. However, the ICD model was shown to be most accurate in the middle of the process. Interestingly, the ensemble was frequently associated with smaller errors than those of the individual models. In the next section, we provide a more comprehensive discussion about this pattern, evaluating how our ensemble compares to other methodologies proposed in the literature.

### Validation of the ensemble

To complete the analysis, we discuss how our conditional trimmed mean ensemble performed against other criteria for combining forecasts. As we forced our predictions to include the ICD compartment model regardless of the value of its predictions, it is possible that our ensemble might lead to a worse performance than those of other criteria that are not subject to this restriction. By design, we were willing to sacrifice precision to gain interpretability, but it is worth exploring whether our predictions were deteriorated by considering this interpretability constraint.

**Fig 6:**
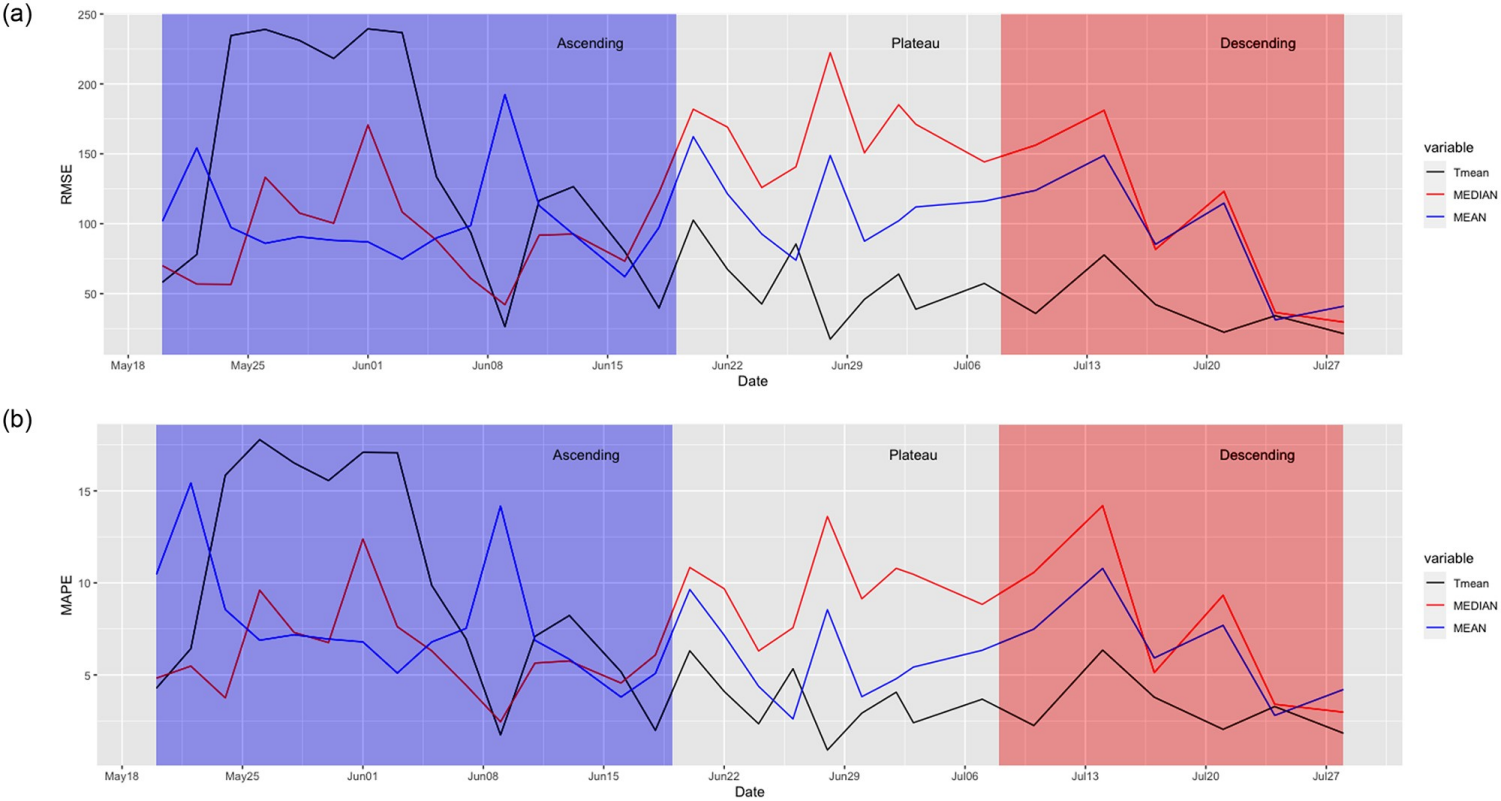
Forecasting errors by ensemble type across iterations. RMSEs are the upper panel and MAPE are in the bottom panel.

Fig 6 displays the root mean square error (RMSE) and mean absolute percentage error (MAPE) for our conditional trimmed mean, along with those of two other commonly used ensembles: the *mean* and the *median* forecasts. For simplicity, in these series, we only report the errors for the whole forecasting horizon with no distinction between the first and second weeks. Instead, in these plots, we highlight three stages depending on whether the series of ICU occupancy exhibited positive, neutral or negative trends; we label them *ascending*, *plateau* and *descending* phases, respectively. Although the definition of the exact time when the series changes its slope is somewhat discretionary (in this exercise, the second phase starts on June 23rd, and the third phase is determined to start on July 8th.), this qualitative decomposition helps us understand the role of the compartmental model in the forecasts. Summaries of the comparisons between the ensemble criteria are displayed in Table 3, where we report the RMSEs and MAPEs for all ensembles and break them down into the three aforementioned stages.

**Table 3:** Error metrics per combination across iterations.

Metric	Tmean	Median	Mean
RMSE (overall)	96.91	115.81	102.92
*Ascending*	140.90	97.29	105.50
*Plateau*	52.37	163.65	106.82
*Descending*	38.95	101.38	90.85
MAPE (overall)	6.78	7.53	6.97
*Ascending*	9.87	6.49	7.94
*Plateau*	3.22	9.55	5.38
*Descending*	3.26	7.60	6.48

From Table 3, we observe that forcing the inclusion of the ICD model did not induce any deterioration in the forecasting precision, and our ensemble forecast exhibited the smallest prediction errors overall. The comparisons by stage shed further light on understanding the performance of the trimmed mean approach.

In the early stages of the pandemic, our trimmed mean criteria were outperformed by the standard mean and median ensembles. However, after a few iterations, our predictions consistently exhibited the smallest errors. This result can be explained by the fact that the ICD model produced prediction errors with opposite signs that canceled out the errors induced by other models. We believe that feeding the model structural information about the clinical evolution of COVID-19 patients can provide a useful forecasting signal and provide additional support for the convenience of using combined forecasts.

## Nontechnical summary of the methodology and implementation

The epidemiological literature has offered a variety of tools for understanding the dynamics of infectious diseases. In this study, we built upon these epidemiological models, and we tailored them with the specific goal of producing accurate forecasts of ICU utilization, as these has been critical components for mitigating the negative impacts of the COVID-19 pandemic. In this regard, there are three key conditions that differentiate our forecast from traditional epidemiological models.

1. As we only focused on patients who required hospital resources in the short term, instead of forecasting the evolution of the pandemic through the reproduction number R, we directly used the number of symptomatic cases. This information is readily available and easy to process. More importantly, the usage of the actual number of symptomatic cases instead of a projection of the infections had a material impact in terms of improving the forecasting accuracy.
2. We were specifically interested in characterizing ICU utilization; therefore, our model was tailored to capture the most relevant dynamics of this problem. These include the persistence of bed utilization and flexible distributions for the duration of the stay of each patient. These dynamics can be captured by two simple conservation law equations that indicate the new daily requirements of beds and the number of discharged patients. The rates at which customers arrive and leave the ICU can be derived from clinical sources, or they can be estimated from the data as we do in our application.
3. In our model, we combined the standard epidemiological approach with time series and machine learning models that bring additional flexibility to the forecast. Importantly, our results indicate that this additional flexibility is critical to obtain highly precise estimates. This is because standard epidemiological models do not properly capture dynamic variations in how patients evolve during critical care. This is particularly relevant for a new disease for which medical teams are continuously learning about improved treatments. Methodologically speaking, we show that the combination of different models can be achieved through a simple linear combination of forecasts.

Since epidemiological models do not incorporate detailed modeling of the dynamics of ICU requirements, they tend to have large forecasting errors. For the case of Chile, even the most sophisticated compartment models exhibited prediction errors that were up to three times larger than what we reported here (http://covid-19vis.cmm.uchile.cl/forecast). Furthermore, these models are rather sensitive to the underlying assumptions about the infection rates and can present more than a 500% difference between their conservative and pessimistic scenarios (http://www.saludpublica.uchile.cl/noticias/163921/informe-covid-19-chile-al-31052020).

Our numerical analysis indicates that the most classical epidemiological approach by itself produces large forecasting errors. In fact, the compartment model generated predictions with a mean error rate of 13.4% (sd of 7.75), while the methodology we proposed led to a mean error rate of 4.11% (sd of 2.52). Some of the time series and machine learning models performed reasonably well, but they failed to anticipate changes in trends. The combined forecast in general produces the most accurate predictions and it correctly anticipates when the number of ICU beds will decrease. Thus, our analysis demonstrates that a simple combination of different forecasts can generate much better predictions in the context of planning emergency resources than those of single models.

Our model may be implemented by health authorities rather easily. Indeed, the logic of our compartment model can be summarized into two flow conservation equations, and the time series and machine learning models can be estimated using standard statistical packages. All forecasts can be combined using a linear combination of the individual forecasts. To facilitate the implementation of the method for other countries or regions, in S4 Table, we provide a summary of the methodological steps and data requirements (which in most countries are already available).

## Conclusions

In this research, we proposed a methodology to produce short-term forecasts for ICU beds in the context of the COVID-19 epidemic in Chile. Our algorithm is based on an ensemble method that combines autoregressive neural networks, artificial neural networks and a compartment model to generate our best prediction of ICU utilization for a time horizon of fourteen days. This algorithm captures the epidemiological dynamics of the disease with a compartmental model and is complemented by time-series models that capture short-term changes in the clinical parameters. This approach resulted in very accurate predictions, with a mean error rate of 4% for the first week and 9% for the second week. An analysis of the performance over time indicates that, in relative terms, the proposed model produced larger errors earlier in the process. This can be explained by the fact that in the early stages of the pandemic, each individual model had less data to learn from. However, we believe that a more fundamental reason is that after a few iterations, different models produced complementary results; therefore, the trimmed mean we used to ensemble the forecast generated a better forecast than that of any single model in isolation. Hence, every model contributed a different key signal that increased the accuracy of the ICU bed predictions in most of our reports. In this regard, the inclusion of a compartmental model helped to generate highly precise predictions, despite being the least accurate single model overall.

In terms of the application, the reports we made publicly available were a very useful tool for anticipating the availability of critical resources in hospitals. We generated consistent information to characterize the progression of the pandemic, providing health officials with a data-driven tool to make quick decisions about ICU planning. These reports enabled the Ministry of Health to implement a progressive increase in the number of beds, and this resulted in more than doubling the capacity in the most congested regions. We heard from health and science authorities and from SOCHIMI how these forecasts were useful for letting them know what was coming and so they could better focus their resources and efforts across the country. Importantly, the messages we were sending were well received because, following our interactions with authorities, we tailored the reports to ease communications.

We are confident that our model contributed to better planning during a critical situation where the lives of many were at risk. However, as the COVID-19 pandemic is still a major threat in many countries around the world, we consider it important to discuss potential ideas to further improve the methodology. In our work, we used the data that were available and that we identified as having predictive power. However, the use of additional disaggregated data is likely to further improve the forecasting accuracy. For example, more detailed information on patient demographics and medical histories could further help to identify what fraction of patients might require mechanical ventilation and thus provide more detailed guidelines about focused mitigation policies.

The proposed methodology can also be improved by adding additional forecasting methods into the pool of models. Although we used a wide variety of models, there are others that we did not try. For example, the recently developed *prophet* forecasting model [61] has been shown to produce proficient predictions for the number of active cases [62]. Our methodology could benefit not only from the addition of more forecasting models but also the addition of other ensemble criteria. For example, recent studies have shown that combining forecasts through ordinary least squares and least absolute deviations can lead to further improvement in the ensemble [63].

To produce our predictions, we treated different regions independently. Although this is a reasonable assumption for the case of Chile where commuting between regions was limited, it might not be a good assumption when replicating our work in other geographies. In such cases, a hierarchical model allowing for spatial correlation might be more appropriate [16]. Finally, in our work, we focused on forecasting the demand for ICU beds with no comprehensive exploration of the underlying mechanisms. A detailed analysis of the parameter estimates could help to understand critical factors are accelerating or decelerating the use of critical resources.

## Supporting information

S1 Table Implementation details. List of libraries and the corresponding parameters used (optional).
(PDF)

S2 Table Model performances in other regions. Historical MAPE per Model—Valparaíso Region.
(PDF)

S3 Table Frequency of selection for each model. Selection Frequency per Model across Iterations in Chile.
(PDF)

S4 Table Implementation guidelines sequence of steps to implement the proposed methodology. (PDF)

S1 Fig Examples of reports for different regions. In the following three figures, we display the summaries of the forecasts for all regions in the country and the detailed plots for the most populated regions of Valparaíso and Bíbio.
(TIF)
