# Supplementary material for: COVID-19: Short-term forecast of ICU beds in times of crisis
Source: PLoS One. 2021 Jan 13;16(1):e0245272. doi: 10.1371/journal.pone.0245272 (PMC7806165; doi:10.1371/journal.pone.0245272)
Supplement: S1 Table — List of libraries and the corresponding parameters used (optional). (PDF) [file pone.0245272.s001.pdf]

S1 Table. Implementation Details. List of libraries and the corresponding parameters used (optional).

Model	R package	Reference	Function	Parameters
ARIMA ARIMAX	forecast	(44)	auto.arima()	allowdrift = TRUE<br>allowmean = TRUE<br>seasonal = FALSE<br>biasadj = TRUE<br>lambda = “auto“
TBATS	forecast	(44)	tbats()	use.damped.trend = TRUE<br>use.trend = TRUE<br>use.arma.errors = TRUE<br>biasadj = TRUE
GMDH	GMDH	(53)	fcast()	layer = 15
MLPR	nnfor	(64)	mlp()	reps = 15<br>difforder = NULL<br>xreg.lags = list(7, 7, 11, 11)<br>hd = c(5, 10, 10, 5)
ELM	nnfor	(64)	elm()	reps = 15<br>difforder = NULL<br>xreg.lags = list(7, 7, 11, 11)<br>direct = TRUE<br>type = “lasso“

Reference

64. Kourentzes N. nnfor: Time Series Forecasting with Neural Networks; 2019. Available from: https://CRAN.R-project.org/package=nnfor.
