# Supplementary material for: COVID-19: Short-term forecast of ICU beds in times of crisis
Source: PLoS One. 2021 Jan 13;16(1):e0245272. doi: 10.1371/journal.pone.0245272 (PMC7806165; doi:10.1371/journal.pone.0245272)
Supplement: S2 Table — Historical MAPE per Model—Valparaíso Region. (PDF) [file pone.0245272.s002.pdf]

S2 Table. Model performances in other regions. Historical MAPE per Model - Valparaíso Region

	1rst week									2nd week								
Date	Ensemble	MEDIAN	MEAN	ARIMAX	MLPR	ELM	TBATS	GMDH	ICD	Ensemble	MEDIAN	MEAN	ARIMAX	MLPR	ELM	TBATS	GMDH	ICD
2020-05-20	23.27	23.06	33.43	29.01	22.90	30.59	22.94		13.77	50.90	54.78	58.85	62.64	54.68	62.17	54.04		24.11
2020-05-22	35.85	40.83	47.49	41.46	36.75	47.57	41.15		26.85	51.70	62.50	62.61	62.58	58.06	68.92	62.69		27.75
2020-05-24	23.80	27.09	36.60	32.98	25.90	28.03	21.83		20.39	25.97	34.40	40.92	45.69	32.40	36.84	22.67		14.48
2020-05-26	9.13	9.88	21.48	10.11	10.81	15.01	4.16		7.40	15.13	11.30	10.62	11.47	46.07	12.38	33.74		12.42
2020-05-28	7.68	8.45	14.76	8.56	29.58	13.88	7.58		7.18	14.97	9.77	12.45	9.73	117.12	8.33	35.30		23.17
2020-05-30	12.75	12.35	11.76	12.05	38.68	10.65	24.10		16.25	32.30	29.81	32.05	29.71	121.03	21.93	74.11		48.13
2020-06-01	24.72	19.73	13.04	19.44	55.60	17.53	30.53		31.37	41.87	31.87	33.15	31.83	133.56	20.02	58.89		56.74
2020-06-03	15.94	11.97	3.81	11.85	19.11	6.71	15.96		26.08	22.84	19.91	6.86	19.91	24.54	4.69	29.51		42.86
2020-06-05	11.13	6.31	6.20	6.51	12.20	4.53	5.17		23.72	18.80	9.23	4.19	14.89	3.71	3.44	8.43		56.28
2020-06-07	6.24	3.51	11.12	3.78	3.81	4.35	4.20		30.77	26.73	4.83	4.85	4.23	9.92	5.50	5.85		105.94
2020-06-09	5.12	3.32	12.63	3.43	3.62	3.72	4.80		17.33	14.03	6.46	6.94	4.61	11.72	6.08	10.04		67.59
2020-06-11	17.54	11.27	14.47	8.22	17.55	6.30	9.07	13.62	35.34	23.47	10.99	17.56	9.44	18.19	3.50	5.46	18.00	61.54
2020-06-13	8.00	5.24	5.73	8.89	4.24	5.24	4.08	8.79	27.08	11.45	3.63	6.27	10.14	5.45	3.63	11.49	21.63	55.30
2020-06-16	4.40	5.16	4.88	4.78	4.51	8.12	11.99	6.87	9.11	1.90	2.01	1.94	2.11	20.69	10.90	20.71	9.91	22.59
2020-06-18	6.23	5.19	6.16	4.65	7.33	9.05	10.61	8.44	4.81	5.24	4.47	4.09	5.61	8.06	9.73	14.73	11.95	4.40
2020-06-20	6.34	7.24	7.23	7.91	10.02	3.12	5.32	5.05	12.41	8.15	12.21	13.50	18.28	27.98	3.31	9.57	7.60	12.74
2020-06-22	3.17	2.35	2.37	1.83	4.70	2.61	2.66	2.34	6.89	3.30	4.92	4.77	9.87	8.40	2.18	2.08	2.75	5.90
2020-06-24	4.07	3.17	2.86	6.72	6.04	3.34	5.07	6.71	2.34	4.29	4.55	2.85	22.01	8.38	3.17	4.64	9.60	5.73
2020-06-26	5.10	6.42	7.91	11.17	12.90	6.38	5.50	5.66	3.04	8.47	12.00	15.34	24.12	25.03	12.00	7.86	10.85	4.59
2020-06-28	6.62	8.89	9.44	10.54	17.78	8.88	7.07	6.14	4.38	8.57	14.91	15.53	20.71	32.75	14.91	7.97	9.66	3.68
2020-06-30	2.76	2.59	2.47	5.25	4.91	4.36	2.87	2.58	2.75	7.38	8.11	8.57	19.24	8.36	14.60	5.46	8.11	2.86
2020-07-02	5.98	5.49	4.93	3.72	6.67	3.48	7.68	5.59	5.44	2.24	10.09	7.04	16.77	10.09	14.43	2.81	2.69	4.71
2020-07-03	3.96	4.43	4.28	8.08	4.91	4.43	3.98	3.90	5.08	9.96	17.23	15.02	31.52	17.23	19.47	7.47	9.30	6.16
2020-07-07	12.45	14.51	12.68	16.47	17.58	14.51	13.21	7.17	4.50	24.70	31.91	27.29	39.77	30.97	35.04	30.80	14.98	2.62
2020-07-10	3.02	5.59	4.51	6.19	3.73	6.90	5.57	4.43	3.84	10.68	21.46	16.99	25.24	13.76	26.36	21.46	14.45	6.96
2020-07-14	3.12	5.95	4.97	9.15	5.90	6.90	4.46	5.55	5.86	8.15	16.77	15.32	27.04	9.88	22.88	15.14	16.77	9.19
2020-07-17	6.12	10.57	8.69	11.98	6.91	11.06	8.36	10.56	1.75	8.84	19.83	17.37	30.52	7.96	23.82	16.62	19.83	9.02
2020-07-21	3.19	5.31	4.49	4.42	6.56	5.35	5.06	7.48	5.82	7.80	13.67	10.29	10.94	19.72	15.43	13.61	8.33	17.56
2020-07-24	1.70	3.22	2.68	2.38	6.03	5.24	3.67	9.44	11.02	3.77	10.23	6.52	8.04	10.69	10.78	11.91	18.27	21.89
2020-07-28	2.13	2.47	2.34	3.25	3.06	5.72	2.45	4.29	5.80	7.25	12.39	12.07	12.58	10.84	19.10	11.48	23.69	5.91
min	1.70	2.35	2.34	1.83	3.06	2.61	2.45	2.34	1.75	1.90	2.01	1.94	2.11	3.71	2.18	2.08	2.69	2.62
max	35.85	40.83	47.49	41.46	55.60	47.57	41.15	13.62	35.34	51.70	62.50	62.61	62.64	133.56	68.92	74.11	23.69	105.94
mean	9.38	9.39	10.85	10.49	13.68	10.12	10.04	6.56	12.61	16.03	16.88	16.39	21.37	30.24	17.19	20.55	12.55	24.76
std	8.23	8.50	10.81	9.23	12.71	9.79	9.29	2.78	10.25	13.61	14.39	15.31	15.56	34.75	16.06	19.15	5.97	25.56
