# Supplementary material for: COVID-19: Short-term forecast of ICU beds in times of crisis
Source: PLoS One. 2021 Jan 13;16(1):e0245272. doi: 10.1371/journal.pone.0245272 (PMC7806165; doi:10.1371/journal.pone.0245272)
Supplement: S3 Table — Selection Frequency per Model across Iterations in Chile. (PDF) [file pone.0245272.s003.pdf]

S3 Table. Frequency of selection for each model. Selection Frequency per Model across Iterations in Chile

ZONE	ARIMA	ARIMAX	MLPR	ELM	TBATS	GMDH	ICD
Country	9,62	49.79	61.09	53.56	70.29	51.88	100.00
Metropolitan	20.00	40.00	60.00	56.67	73.33	60.00	100.00
Other Regions	8.13	51.20	61.24	53.11	69.86	50.72	100.00
