# Supplementary material for: COVID-19: Short-term forecast of ICU beds in times of crisis
Source: PLoS One. 2021 Jan 13;16(1):e0245272. doi: 10.1371/journal.pone.0245272 (PMC7806165; doi:10.1371/journal.pone.0245272)
Supplement: S4 Table — (PDF) [file pone.0245272.s004.pdf]

S4 Table. Implementation Guidelines Sequence of steps to implement the proposed methodology.

**Step**	**Description**
1: Data Preparation	Import data.
	Define the predictive variables and determine the length of the lags for each variable.
	Create time series for ICU utilization and the predictive variables.
2: Forecasting	Select the machine learning models & autoregressive models to use.
	Check if the forecasting models require data normalization.
	Check if the forecasting models use exogenous variables.
	Estimate the forecasting models using cross validation.
	For each model, select the instance that minimizes the error for the holdout sample.
3: ICU Simulation	Determine the number of new symptomatic cases that will require ICU. This proportion can be computed from clinical records or from the observed fractions of the previous days.
	Determine the time at which these new symptomatic cases will require ICU beds. The delay between the identification of new cases and the use of ICU beds can be computed from clinical records or from the observed durations on previous days.
	Determine the time at which these new symptomatic patients will be discharged from the ICU. This time can be computed from clinical records or from the observed durations on the previous days.
4: Combined Forecasting	Generate a combined forecast by an ensemble of the models generated in Steps 2 and 3. We suggest discarding the models with the most extreme predictions.
5: Report and Evaluation	Evaluate the performance of the combined forecast by comparing it to actual ICU occupancy.
	Prepare reports summarizing the results for decision makers.
