# Supplementary figures and images for: COVID-19: Short-term forecast of ICU beds in times of crisis
Source: PLoS One. 2021 Jan 13;16(1):e0245272. doi: 10.1371/journal.pone.0245272 (PMC7806165; doi:10.1371/journal.pone.0245272)

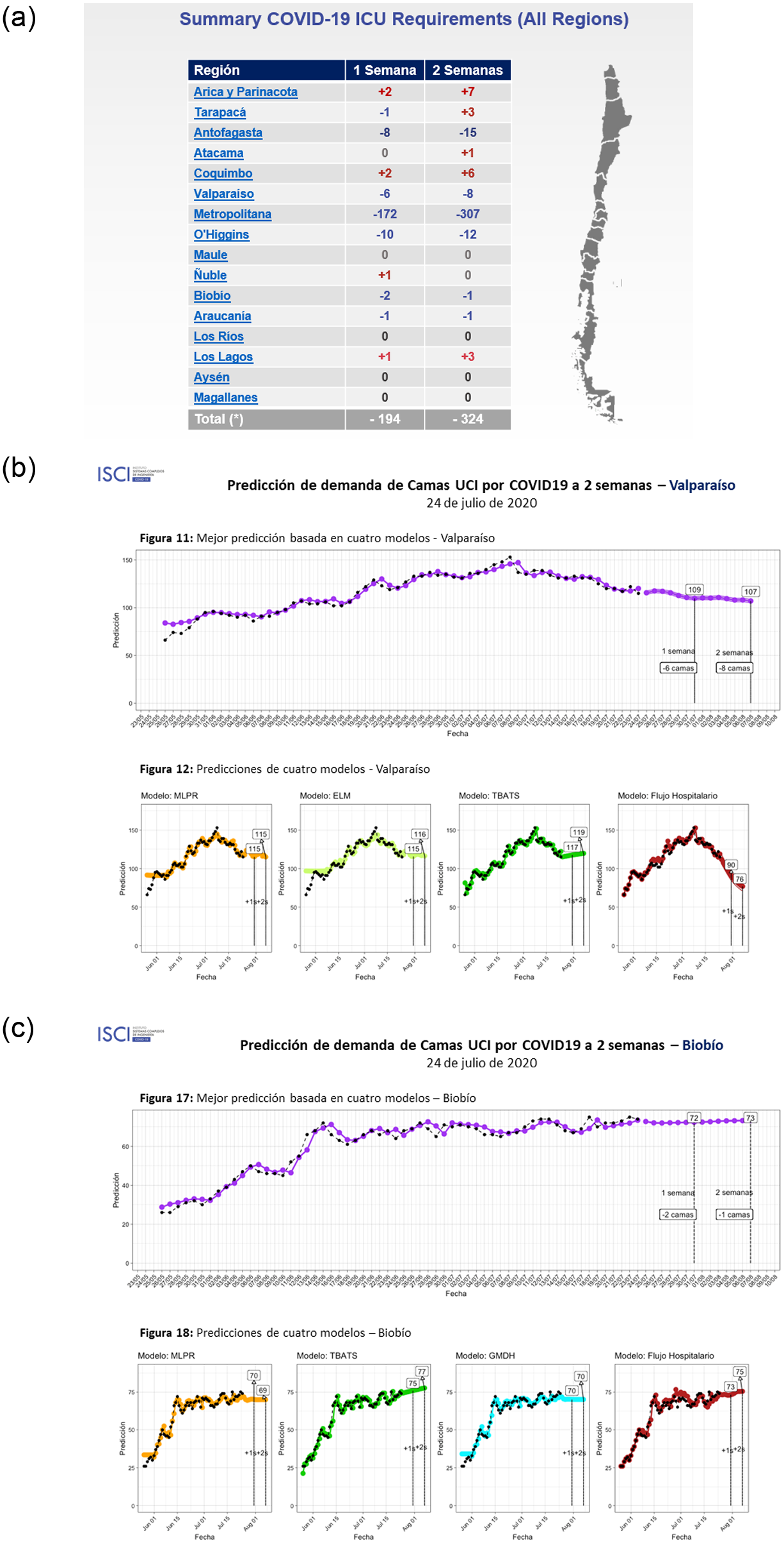

Supplement: S1 Fig — In the following three figures, we display the summaries of the forecasts for all regions in the country and the detailed plots for the most populated regions of Valparaíso and Bíbio. (TIF) [file pone.0245272.s005.tif]
